# Can cervical vascular ultrasound combined with transcranial Doppler ultrasound accurately diagnose cerebral infarction? A protocol for a systematic review and meta-analysis

**DOI:** 10.1097/MD.0000000000019997

**Published:** 2020-05-29

**Authors:** Li-wei Qin, Chen Wang, Xiu-juan Feng, Xiao-hui Wang, Li-hong Qin, Christina Weeks

**Affiliations:** a Department of Physical Diagnosis; b Second Ward of Neurology Department; c First Ward of Neurology Department, First Affiliated Hospital of Jiamusi University, Jiamusi, China; d Medical School, University of Edinburgh, Edinburgh, EH8 9AG, UK.

**Keywords:** case-controlled study, cerebral infarction, cervical vascular ultrasound, sensitivity, specificity, transcranial Doppler ultrasound

## Abstract

**Background:**

The purpose of this study is to investigate the impact of cervical vascular ultrasound (CVU) combined transcranial Doppler ultrasound (TDU) in the diagnosis of cerebral infarction (CI).

**Methods:**

The following electronic databases will be sought from PUBMED, EMBASE, Cochrane Library, PSYCINFO, Web of Science, Allied and Complementary Medicine Database, WANGFANG, VIP database, and China National Knowledge Infrastructure. The search period will cover from the initial indexing to March 1, 2020 without restrictions of language and publication status. All case-controlled studies which identifying the impact of CVU combined TDU in the diagnosis of CI will be considered. Two authors will independently perform the whole process of study selection, data extraction, and quality assessment, respectively. If any disagreements occur between two authors, we will invite a third experienced author to help solve them through discussion. Quality Assessment of Diagnostic Accuracy Studies tool will be used to check study quality, and RevMan V.5.3 software and Stata V.12.0 software will be utilized to carry out statistical analysis.

**Results:**

This study will summarize the most recent evidence that focusing on the impact of CVU combined TDU in the diagnosis of CI.

**Conclusion:**

This study will provide helpful evidence to determine whether CVU combined TDU is an accurate diagnosis tool for CI or not.

**Systematic review registration:**

PROSPERO CRD42020171367.

## 1 Introduction

Cerebral infarction (CI) is the most common cerebrovascular disorder seen in stroke,^[1–4]^ with the highest morbidity ∼70%.^[5]^ It has been estimated that it accounts for about 60% to 80% of stroke.^[6–9]^ It manifests as dizziness, severe headache, paralysis or numbness of face or limbs, speech difficulty, and even death.^[10–14]^ Many risk factors can result in CI, such as hypertension, diabetes mellitus, and cardiovascular disease.^[15–19]^ Thus, it is very important to diagnose CI at early stage for early managements.

Previous studies have reported that cervical vascular ultrasound (CVU) combined transcranial Doppler ultrasound (TDU) can be utilized to diagnose CI.^[20–34]^ However, there are still inconsistent results, and no systematic review has specifically explored this issue. Therefore, this study will investigate the impact of CVU combined TDU in the diagnosis of CI.

## 2 Methods and design

### 2.1 Protocol registry

This study is reported based on the Preferred Reporting Items for Systematic Review and Meta-Analysis Protocols (PRISMA-P) Statement,^[35]^ and it has been registered on PROSPERO database (CRD42020171367).

### 2.2 Inclusion criteria for study selection

#### 2.2.1 Type of studies

We will include all case-controlled studies that explored the impact of CVU combined TDU in the diagnosis of CI. We will exclude any other studies, such as animal studies and reviews.

#### 2.2.2 Type of participants

All adult participants (over 18 years old) who were clinically diagnosed with computed tomography or magnetic resonance imaging-proven CI for inclusion. No gender and race will be considered in this study.

#### 2.2.3 Type of index test

Index test: All participants who received CVU combined TDU diagnosis will be included in the experimental group.

Reference test: All participants who were diagnosed by computed tomography or magnetic resonance imaging-proven CI will be considered as controls.

#### 2.2.4 Type of outcome measurements

The primary outcomes include sensitivity and specificity. The secondary outcomes comprise of positive likelihood ratio, negative likelihood ratio, and diagnostic odds ratio.

### 2.3 Data sources and search strategy

#### 2.3.1 Electronic searches

The following electronic databases will be searched in PUBMED, EMBASE, Cochrane Library, PSYCINFO, Web of Science, Allied and Complementary Medicine Database, WANGFANG, VIP database, and China National Knowledge Infrastructure. The search period will cover from the initial indexing to the March 1, 2020 with no limitations of language and publication status. We will include all case-controlled studies which assessed the impact of CVU combined TDU in the diagnosis of CI. The sample of search strategy for PUBMED is presented in Table 1. In addition, we will also adapt similar search strategies to other electronic databases.

**Table 1:**
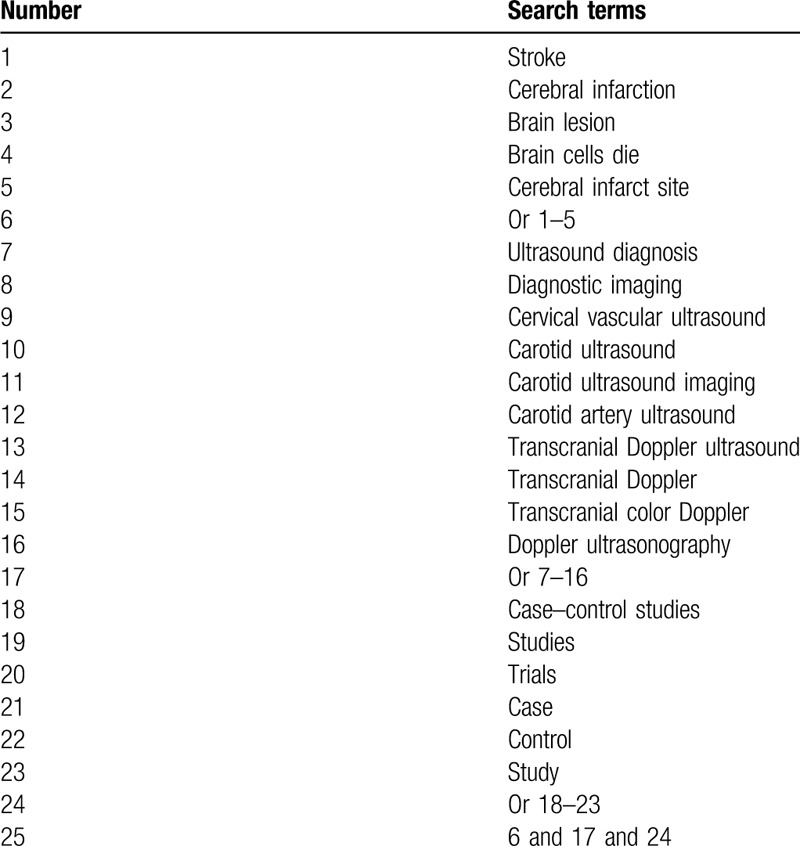
Search strategy utilized in PUBMED database.

#### 2.3.2 Other resources

We will also search grey literature search of targeted clinical trial registries, conference abstracts, dissertations, and reference lists of related reviews.

### 2.4 Study selection and data collection

#### 2.4.1 Study selection

There are two stages in the whole process of study selection by two independent authors. At the first stage, the titles and abstracts of all identified studies will be read according to the predefined eligibility criteria. All unrelated studies and duplicates will be excluded. At the second stage, the full-text articles of all potential citations will be evaluated against full inclusion criteria. Divergences between two authors will be solved through consensus by a third author. The study selection process will be reported using a detailed flow chart. A list of excluded records alongside the rationale of their exclusion will be presented in an additional file.

#### 2.4.2 Data extraction

Before data extraction, a standardized data extraction sheet will be created for study information collection. Two authors will independently carry out data extraction. Any disagreements will be resolved by discussion with the help of a third author. Information from each included study consists of publication characteristics (e.g., first author, year of publication, and title), patient information (e.g., race, gender, and inclusion and exclusion criteria), study design traits (e.g., trial setting, trial design, sample size, and dropouts), index and reference tests, and outcome indicators (e.g., reported outcomes and summary data related to the index and reference tests). If missing or insufficient information occurs during the period of data collection, we will contact original authors to obtain it.

### 2.5 Study quality assessment

We will examine study quality using The Quality Assessment of Diagnostic Accuracy Studies tool.^[36]^ Two independent authors will check study quality for each eligible study. Any objections will be settled down by a third author through discussion.

### 2.6 Assessment of heterogeneity

Statistical heterogeneity across the included studies will be identified using *I*^2^ test. *I*^2^ ≤ 50% presents acceptable heterogeneity, and we will use Mantel-Haenszel fixed-effects model to pool the data. Otherwise, *I*^2^ > 50% shows significant heterogeneity, and we will utilize Mantel-Haenszel random-effects model to synthesize the data.

### 2.7 Statistical analysis

#### 2.7.1 Data synthesis

This study will utilize RevMan V.5.3 software and Stata V.12.0 software to pool and to analyze the outcome indicators, which will be presented as descriptive statistics and 95% confidence intervals. A descriptive forest plot and a summary receiver operating characteristic plot will be undertaken if necessary. We will carry out meta-analysis if we will find acceptable heterogeneity across the sufficient eligible studies with similar study and patient characteristics, index test and reference test, and outcome indicators. Otherwise, we will conduct subgroup analysis to identify the sources of significant heterogeneity. If meta-analysis is inappropriate in this study, a narrative summary of findings with supporting tables and figures will be reported and presented.

#### 2.7.2 Subgroup analysis

We will explore subgroup analysis to investigate the sources of significant heterogeneity in accordance with the different types of study characteristics, index test and reference test, and outcome indicators.

#### 2.7.3 Sensitivity analysis

We will carry out a sensitivity analysis to examine the stability of our findings and impact of covariates by eliminating low quality studies.

#### 2.7.4 Reporting bias

Reporting bias will be checked by funnel plots and associated regression test when at least 10 qualified studies are included.^[37]^

### 2.8 Ethics and dissemination

No ethic approval will be used in this study, because it will not collect original data. We will publish this study through a relevant peer-reviewed journal or a conference.

## 3 Discussion

This study will firstly investigate the impact of CVU combined TDU in the diagnosis of CI. We will systematically and comprehensively search more electronic databases and other literature sources to avoid missing potential studies. Two independent authors will perform study selection, data extraction and study quality assessment. We will invite a third author to solve any inconsistencies between two authors. The study quality will be assessed using The Quality Assessment of Diagnostic Accuracy Studies tool.

Although a numerous of studies have reported that CVU combined TDU can help diagnose CI, there are still inconsistent results.^[19–34]^ The findings of this study may provide recommended evidence of CVU combined TDU diagnosis in detection CI, which may benefit both patients and clinicians.

## Author contributions

**Conceptualization:** Li-hong Qin, Chen Wang, Xiu-juan Feng.

**Data curation:** Li-hong Qin, Li-wei Qin, Xiao-hui Wang, Christina Weeks.

**Formal analysis:** Li-hong Qin, Li-wei Qin, Chen Wang, Xiu-juan Feng, Xiao-hui Wang.

**Funding acquisition:** Li-hong Qin.

**Investigation:** Li-hong Qin.

**Methodology:** Li-wei Qin, Chen Wang, Xiu-juan Feng, Xiao-hui Wang.

**Project administration:** Li-hong Qin.

**Resources:** Chen Wang, Xiu-juan Feng, Xiao-hui Wang, Christina Weeks.

**Software:** Li-wei Qin, Chen Wang, Xiu-juan Feng, Xiao-hui Wang.

**Validation:** Li-hong Qin, Li-wei Qin, Chen Wang, Xiao-hui Wang, Christina Weeks.

**Visualization:** Li-hong Qin, Chen Wang, Xiu-juan Feng, Xiao-hui Wang, Christina Weeks.

**Writing – original draft:** Li-hong Qin, Li-wei Qin, Chen Wang, Xiu-juan Feng, Xiao-hui Wang.

**Writing – review & editing:** Li-hong Qin, Li-wei Qin, Xiao-hui Wang, Christina Weeks.
